# Role and therapeutic potential of G-protein coupled receptors in breast cancer progression and metastases

**DOI:** 10.1016/j.ejphar.2015.05.011

**Published:** 2015-09-15

**Authors:** Anukriti Singh, Jessica J. Nunes, Bushra Ateeq

**Affiliations:** a Department of Biological Sciences and Bioengineering, Indian Institute of Technology, Kanpur 208016, Uttar Pradesh, India

**Keywords:** Breast cancer, GPCR, AGTR1, ACE, Metastases

## Abstract

G-protein-coupled receptors (GPCRs) comprise a large family of cell-surface receptors, which have recently emerged as key players in tumorigenesis, angiogenesis and metastasis. In this review, we discussed our current understanding of the many roles played by GPCRs in general, and particularly Angiotensin II type I receptor (AGTR1), a member of the seven-transmembrane-spanning G-protein coupled receptor superfamily, and its significance in breast cancer progression and metastasis. We have also discussed different strategies for targeting AGTR1, and its ligand Angiotension II (Ang II), which might unravel unique opportunities for breast cancer prevention and treatment. For example, AGTR1 blockers (ARBs) which are already in clinical use for treating hypertension, merit further investigation as a therapeutic strategy for AGTR1-positive cancer patients and may have the potential to prevent Ang II-AGTR1 signalling mediated cancer pathogenesis and metastases.

## 1 Introduction

G-protein-coupled receptors (GPCRs), the largest family of cell-surface receptors has been known to play a critical role in the neoplastic transformation of many cancers including hormone-refractory cancers of breast and prostate. Some of the key functions of GPCRs include regulation of cellular motility, growth and differentiation, which play an important role in understanding the biology of cancer (Spiegelberg and Hamm, 2007). In 1986, the discovery of the MAS oncogene, which encodes a typical GPCR, established a direct connection between neoplastic transformation and GPCRs (Young et al., 1986). Many independent studies have shown that GPCRs are over-expressed in variety of cancer types, and contribute in cell proliferation when activated by their respective circulating or locally available ligands (Even-Ram et al., 1998, Rhodes et al., 2009). Furthermore, wild-type GPCRs could also become oncogenic when exposed to an excess of locally produced or circulating agonists such as gastrin-releasing peptide (GRP), endothelin, bradykinin and Ang II (Gutkind et al., 1991, Julius et al., 1989); in addition mutations in the conserved domain of the GPCRs could also trigger oncogenic transformation (Allen et al., 1991). Moreover, the activation of endothelin receptors, bradykinin receptors, the Angiotensin II type I receptors (AGTR1) (de Gasparo et al., 2000, Rhodes et al., 2009) and gastrin-releasing peptide receptors (GRPR) has been implicated in prostate cancer pathogenesis (Daaka, 2004).

## 2 G-protein coupled receptors in breast cancer

The role of GPCRs in breast cancer (BCa) has been explored extensively, for example protease-activated receptor 1 (PAR1) is over-expressed in BCa and is responsible for development of metastases in BCa patients (Hernandez et al., 2009). PAR1 is also known to promote growth and invasion by promoting detachment and migration of the epithelial cancer cells, which is a key step in tumour metastases (Boire et al., 2005, Hernandez et al., 2009). PAR1 also couples to multiple G-proteins (G_q/11_, G_i/o_, G_12/13_) and activates Rho signalling (McCoy et al., 2012), thereby resulting in changes in cytoskeleton structure and cell shape (Austin et al., 2013), suggesting its critical role in BCa metastases. Another GPCR, GPR116, plays an important role in cell adhesion and is found to be a novel regulator of BCa metastasis. GPR116 regulates morphology and cell motility through the Gαq-p63RhoGEF-RhoA/Rac1 pathway. Activated Rho GTPases are known to induce many downstream signalling pathways such as ROCK1/2 during cancer cell migration. Specifically, p63RhoGEF (GEFT), a guanine nucleotide exchange factor (GEF) acts as an effector of the guanine-nucleotide binding protein Gαq, thus linking GPCRs to the activation of the Rho-GTPases. Knockdown of GPR116 in MDA-MB-231, a triple negative, hormone-insensitive and metastatic breast cancer cell line results in significant decrease in cell migration and invasion, suggesting that GPR116 promotes BCa cell migration and invasion via Gαq signalling and p63RhoGEF (a Gαq effector) mediated activation of the RhoA and Rac1 (Tang et al., 2013). Taken together, we speculate that targeting both PAR1 and GPR116 in triple negative breast cancer (TNBC) may hold great promise in targeting these pathways and combating hormone-refractory breast cancer.

Nevertheless, orphan GPCRs represent a highly active area of research that has already led to the identification of many new ligands for previously orphaned GPCRs. One such orphan GPCR is GPR161, a class A rhodopsin family member. GPR161 was found to be overexpressed specifically in TNBC and is also correlated with poor prognosis. Importantly, knockdown of the GPR161 in basal breast cancer cell lines demonstrated inhibition in cell proliferation. GPR161 forms a signalling complex with two scaffold proteins, namely β-arrestin 2 and Ile Gln motif containing GTPase Activating Protein 1 (IQGAP1). Cells overexpressing GPR161 activate mammalian target of rapamycin (mTOR) signalling thereby decreasing IQGAP1 phosphorylation. Conversely, unphosphorylated IQGAP1 binds mTOR which leads to the activation of downstream signals, one of which is phosphorylation of S6, a ribosomal protein (Feigin et al., 2014). Taken together, we anticipate that tumour-specific gene expression and proteome profiles of the tumour tissues and premalignant lesions, in combination with ‘reverse pharmacology’ techniques, will aid in identifying and targeting new orphan GPCRs that may contribute to cancer initiation, progression and metastases.

Another interesting member of the seven-transmembrane-spanning G-protein coupled receptor superfamily is AGTR1, which was prioritized as second ranked meta-outlier by employing a bioinformatics tool named meta-Cancer Outlier Profile Analysis (MetaCOPA) using multiple independent breast cancer profiling studies (Rhodes et al., 2009). As anticipated, HER2/neu was identified as the most significant meta-outlier and AGTR1 as the second most consistently high-scoring gene in BCa, which is also known for its role in Ang II‐dependent vasoconstriction (Luft, 2001, Schmieder et al., 2007). While AGTR1 is found in a variety of normal tissues, increased expression is often observed in the corresponding neoplastic tissues, suggesting that it’s over-expression is involved in carcinogenesis (Marsigliante et al., 1996, Takeda and Kondo, 2001). AGTR1 has also been linked to pancreatic, renal and ovarian cancers (Fujimoto et al., 2001, Miyajima et al., 2002, Rivera et al., 2001, Suganuma et al., 2005, Timmermans, 1999, Uemura et al., 2003) and cancer-related signalling pathways (Amaya et al., 2004, Muscella et al., 2003). AGTR1 is over-expressed in oestrogen receptor positive (ER+) tumours and mutually exclusive with HER2/neu, indicating the possibility that over-expression of these two genes may represent alternative pathways in breast cancer pathogenesis (Ateeq et al., 2009, Rhodes et al., 2009). Most importantly, AGTR1 mediated oncogenic effects could be antagonized by commonly prescribed antihypertensive agents, such as losartan (Rhodes et al., 2009, Timmermans, 1999). It is interesting to note that the BCa prevalence was reported low in hypertensive patients who received angiotensin converting enzyme inhibitors (ACEi) previously, which blocks the conversion of Ang I to Ang II, thereby reducing activation of AGTR1 by Ang II (Lever et al., 1998). Similarly, ARBs have been reported to inhibit cell proliferation and angiogenesis in prostate cancer cells (Uemura et al., 2003). Ang II mediates its complex physiological effects by binding to two pharmacologically distinct receptors; AGTR1 and Angiotensin II Type 2 Receptor (AGTR2) (Timmermans et al., 1992). The stimulatory actions of Ang II on angiogenesis, cell growth, and cell proliferation in tissues are mediated via AGTR1 (De Paepe et al., 2001, Egami et al., 2003) and opposed via AGTR2 (Goto et al., 2002, Silvestre et al., 2002). Moreover, Ang II activates AGTR1, which couples to the heterotrimeric G proteins G_q/11_ (to stimulate phospholipase C mediated calcium mobilization), G_i/o_, G_12/13_ and G_s_, as well as the other monomeric G proteins (de Gasparo et al., 2000). It has been shown that Arhgef1, a RhoA guanine exchange factor is specifically responsible for Ang II-induced activation of RhoA signalling and as a result Jak2 phosphorylates Tyr738 of Arhgef1 (Guilluy et al., 2010). In addition, activated AGTR1 also activates soluble and receptor tyrosine kinases, the mitogen-activated protein kinases (MAPK pathway), the JAK–STAT pathway, the generation of reactive oxygen species and various ion channels (de Gasparo et al., 2000, Hunyady and Catt, 2006, Mehta and Griendling, 2007). Taken together, AngII–AGTR1 signalling pathways play a critical role in the pathogenesis of AGTR1-positive breast and prostate cancer.

## 3 Single nucleotide polymorphisms in AngII–AGTR1 pathway

Recent genome-wide association studies have revolutionized the field of cancer research and led to the identification of numerous single nucleotide polymorphism (SNP), which are associated with increased risk for breast cancer (Easton et al., 2007). For example, germline mutations in *BRCA1* and *BRCA2* could predispose women to BCa, as well as to ovarian cancer (King et al., 2003). Somatic mutations in other genes, such as *p53*, *PTEN*, or *CHEK2*, are also associated with increased risk of BCa (Hirshfield et al., 2010, Weischer et al., 2008). Moreover, an association between the genetic polymorphisms in the 5′ region of *AGTR1* and the increased risk of BCa has been reported among Chinese women. This study also revealed three genetic polymorphisms A168G, C535T, T825A in the 5′ region of *AGTR1*. Individuals harbouring genotypes with one or two copies of these allelic variants were found to be associated with 30% lower risk of BCa as compared to the homozygotes (Koh et al., 2005). Conversely, another independent study showed no significant association between A168G polymorphism of AGTR1 and BCa risk, but demonstrated the significance of AGTR2 SNPs (T1247G and A5235G) as a predictor of BCa in Brazilian women (Molina Wolgien Mdel et al., 2014). Nevertheless, deletion of the 5′ flanking region of *AGTR1* showed 20-fold increase in chloramphenicol acetyltransferase reporter activity, thus confirming the presence of a negative regulatory element(s) in the upstream region of *AGTR1* (Takayanagi et al., 1994). These observations indicate that the genetic variants in the 5′ flaking region of *AGTR1* might be associated with an increase in breast cancer risk. Furthermore, increased frequency of a SNP at 1166 position (A/C transversion) in the 3′ UTR of AGTR1 has been associated with hypertension (Bonnardeaux et al., 1994), cardiac hypertrophy (Osterop et al., 1998), myocardial infarction (Tiret et al., 1994) and increased oxidative stress levels in human heart failure (Cameron et al., 2006).

An association between Angiotensin I converting enzyme (ACE), which converts Ang I into a physiologically active form Ang II and BCa risk has been demonstrated (Lever et al., 1998). The SNP of ACE (A240T and I/D) regulates its level in the plasma, for example homozygotic individuals for D or T alleles have higher ACE levels than in the homozygotic individuals for I or A alleles. Therefore, the individuals with ACE genotype (II or AA) have a lower risk for BCa in comparison to the ones with high activity (DD or TT) alleles (Koh et al., 2003, Koh et al., 2005). Furthermore, a SNP (A1166C) in the *AGTR1* has been associated with higher tumour node metastases (TNM) stage of the BCa as compared to the individuals harbouring A1166A (Namazi et al., 2010). However, in a follow-up study, no association between this polymorphism and three years disease free survival was found (Namazi et al., 2013). Conversely, reduced plasma levels of the ACE were not always observed in the individuals with I or A allele (Freitas-Silva et al., 2004, Haiman et al., 2003), suggesting that the association of ACE genotype with BCa risk depends on the ethnicity of the population. We speculate that the genetic polymorphisms in AngII–AGTR1 pathway may have racial disparity. Therefore, additional studies exploring SNPs in the AngII–AGTR1 pathways are warranted on the populations of different ethnicities. Moreover, a population specific genetic profile could be created for evaluating cancer survival based on prognosis markers, which would eventually help in understanding the differences reported for the BCa incidence and outcomes, based on geography and ethnicity.

## 4 AngII–AGTR1 signalling mediated epithelial-to-mesenchymal transition

**Fig. 1:**
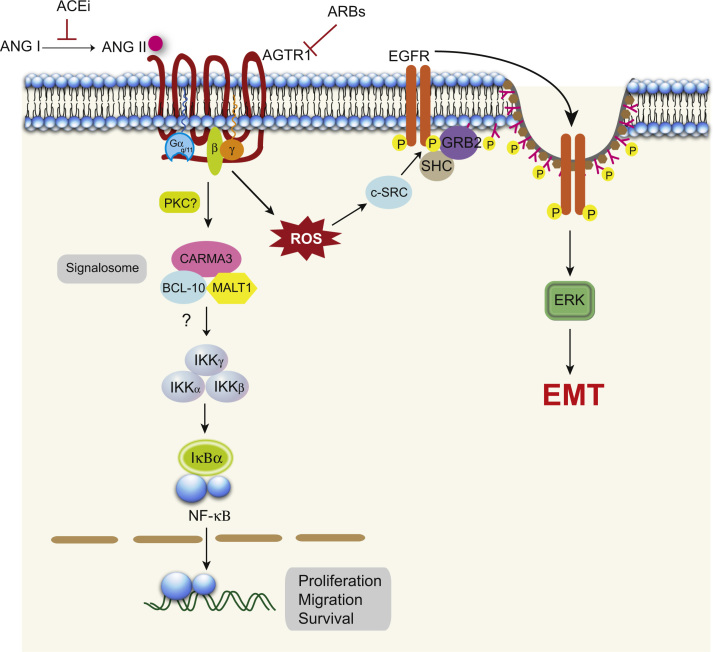
Major AGTR1 signalling pathways linked to cancer cell proliferation, angiogenesis and EMT. AngII activated AGTR1 recruits a CARMA3-Bcl10-MALT1 (CBM) signalosome, which activates NFκB downstream signalling. CARMA3 protein might act as a scaffold in recruiting Bcl 10, MALT 1 and IKKγ, the regulatory subunit of the IKK complex. Wherein MALT1 plays a key role in stimulating IKK activity by K63 linked polyubiquitination utilizing IKKγ as a substrate. The activation of this pathway leads to cell proliferation, survival and migration. AngII activation of AGTR1 also leads to EGFR transactivation via ROS‐ dependent Src kinase activation, phosphorylating EGFR and the adaptor proteins GRB2 and SHC, resulting in prolonged EGFR–ERK signalling. Continuous Ang II stimulation may direct alterations in the gene expression and induce phenotypic change from epithelial-to-mesenchymal transition (EMT).

Various cellular responses such as cell proliferation, differentiation or dedifferentiation are triggered by a variety of external stimuli, which involves the transcriptional regulation in cancer cells through intracellular signalling cascades, including multitude of signalling pathways that activate kinases of the mitogen-activated protein kinase (MAPK) family (Treisman, 1996) either through receptor tyrosine kinase (RTK)- or through GPCR-triggered signals (Faure et al., 1994, Pages et al., 1993, van Biesen et al., 1996). It has been known that AGTR1 hijacks epidermal growth factor receptor (EGFR) signalling machinery, which is critical for the AGTR1 mediated downstream signalling and phenotypic effects, such as cellular hypertrophy and proliferation (Asakura et al., 2002, Eguchi et al., 2001, Mifune et al., 2005). Ang II-induced platelet derived growth factor receptor β (PDGFR-β) and thrombin stimulated insulin-like growth factor-1 receptor (IGF-1R) tyrosine phosphorylation have been reported in primary rat smooth muscle cells (Linseman et al., 1995, Rao et al., 1995), suggesting that transactivation of distinct RTKs might contribute in a cell-type specific manner to GPCR mediated mitogenic signalling. Moreover, Ang II-activated EGFR signalling in renal proximal tubule epithelial cells results mostly by the non-ligand-mediated receptor transactivation mediated by ROS-dependant Src activation, which leads to phosphorylation of both EGFR and Caveolin-1 and their association in the lipid rafts (Fig. 1) (Chen et al., 2012, George et al., 2013). Thus, the constant activation of the EGFR serves as a scaffold for SHC/GRB2-mediated ERK activation, subsequently resulting in the dedifferentiation or epithelial-to-mesenchymal transition (EMT) of renal proximal tubule epithelial cells (Chen et al., 2012). These studies indicate that AngII–AGTR1 prolonged signalling activity in the AGTR1-positive cancers may lead to alterations in gene expression and consequently elicit a phenotypic change to EMT, which promotes aggressive phenotype and distant metastases. Interestingly, using functional siRNA screen of the human kinome, new signalling targets such as *TRIO*, *BMX* or *CHKA* have been revealed, which upon knockdown attenuate tyrosine phosphorylation of the EGFR by Ang II stimulation, but failed to directly stimulate EGFR via EGF, suggesting that these proteins are involved in AGTR1–EGFR transactivation (George et al., 2013). Nevertheless, a deeper and comprehensive understanding of AngII–AGTR1 axis and AGTR1–EGFR crosstalk in the context of AGTR1 positive cancers may direct future studies, which may lead to the development of novel drug targets against these pathways as an alternative to existing cancer therapies.

## 5 Targeting AGTR1 for enhanced drug delivery and improved chemotherapy

Several FDA-approved ARBs, which are orally active have been synthesized and are widely prescribed for the treatment of hypertension, such as losartan, irbesartan, olmesartan, candesartan, valsartan and telmisartan. Previously, we have shown that ectopic over-expression of AGTR1 in immortalized normal breast epithelial cells, confers an invasive phenotype upon AngII stimulation, which was attenuated by losartan (Rhodes et al., 2009). Losartan has also been shown to inhibit many growth factors, including vascular endothelial growth factor (VEGF) (Arrieta et al., 2005). Interestingly, preclinical mice experiments with control or AGTR1 overexpressing breast cancer xenografts showed differential sensitivity to losartan treatment, resulting in 30% decrease in tumour growth in AGTR1 overexpressing group, whereas no effect was observed in the control group (Rhodes et al., 2009). Another ARB, candesartan has been reported to reduce lung metastases, vascularization and tumour growth in sarcomas and melanoma xenografts (Fujita et al., 2002; Egami et al., 2003). On the other hand, telmisartan which is a structurally unique ARB, renders more effective inhibition of the AGTR1 mediated pro-tumorigenic effects and the unique structural characteristics provide partial agonistic response for a member of nuclear receptor family peroxisome proliferator-activated receptor-γ (PPARγ) (Benson et al., 2004). Losartan and telmisartan, both demonstrate higher tissue penetration as compared to candesartan (Michel et al., 2013), which could be a possible reason for selecting Losartan over other ARBs for a pancreatic cancer clinical trial study (Chauhan et al., 2013).

AGTR1 employs the CARMA3-Bcl10-MALT1 (CBM) signalosome for the activation of NF-κB signalling in endothelial and vascular smooth muscle cells (VSMC), thus inducing pro-inflammatory signalling in the vasculature that may lead to atherosclerosis (McAllister-Lucas et al., 2010). Interestingly, Bcl10 deficient mice failed to develop Ang II‐dependent atherosclerotic lesions and abdominal aortic aneurisms (McAllister-Lucas et al., 2010). The decreased rate of arthrosclerosis has also been associated with decrease in the expression of NF-κB responsive genes (Surmi and Hasty, 2010). Interestingly, siRNA mediated knockdown of CARMA3 in AGTR1 over-expressing immortalized VSMC cell lines, showed no response to Ang II dependent ERK activation or TNFα‐dependent pIκB generation (McAllister-Lucas et al., 2010), suggesting that CARMA3 protein might act as a scaffold in recruiting Bcl10, MALT1 and IKKγ, the regulatory subunit of the IKK complex (Stilo et al., 2004). These observations gain much more importance in light of the recent discovery of an inhibitor of MALT1 protease, a component of the signalosome that is enzymatically active and communicates downstream with NF-κB signalling (Rebeaud et al., 2008). Hence we can infer that the CBM signalosome may play a major role in AGTR1 mediated breast cancer pathogenesis, and MALT1 or Bcl10 inhibitors might prove as promising targets.

On another note, proliferating cancer cells are known to consecutively create a new solid substance comprising cells and matrix components, which generate radial and circumferential solid stress (Kharaishvili et al., 2014). This stress in the growing tumour collapses blood vessels and limits perfusion resulting in extensive hypoxia and impaired drug delivery (Griffon-Etienne et al., 1999, Janmey and McCulloch, 2007, Padera et al., 2004). As a result, cancer patients with low tumour perfusion show poor chemotherapy responses and shorter survival versus patients with high perfusion (Park et al., 2009, Sorensen et al., 2011). Interestingly, AGTR1 inhibitors/antagonists have been known to increase vessel perfusion through vascular decompression, thereby reducing stromal activity and production of matrix components responsible for compression. Likewise, AGTR2 agonists or inhibitors of downstream signalling through TGF-β1, CCN2 or ET-1 have been known in reducing solid stress to enhance chemotherapy and overcome challenges associated with chemotherapies (Chauhan et al., 2013). Furthermore, AGTR1 signalling plays an important role in increasing VEGF expression by Cancer Associated Fibroblasts (CAFs) (Fujita et al., 2005), thus both ACE and AGTR1 inhibitors could be used to target VEGF expression and angiogenesis (Suganuma et al., 2005, Yoshiji et al., 2001).

ACE inhibitors (ACEi), which have been successfully used as antihypertensive drugs for the past 20 years, are now being investigated for their possible role as anticancer compounds (Lindberg et al., 2004). Interestingly, epidemiological study suggests that the long-term treatment of ACEi such as captopril, lisinopril and enalapril has reduced the incidence of lung and breast cancer (Lever et al., 1998). However, other epidemiological studies were not in concordance with these results and showed that ACEi treatment had no significant effect on cancer (Friis et al., 2001, Li et al., 2003, Lindholm et al., 2001). One possible explanation might be that the latter studies enroled older patients who underwent treatment for shorter duration. Moreover, use of different ACEi on diverse population of patients along with variability in the dosage, duration of drug prescription as well as patient compliance might be the possible reasons for the contradictory results (Deshayes and Nahmias, 2005). Taken together, ACE and AGTR1 blockers could be used as an adjuvant therapy along with established chemotherapeutic drugs to further potentiate the anti-cancer effects of the conventional cancer therapies. Specifically, AngII–AGTR1 axis could be further explored as a potential therapeutic target for treating AGTR1 positive cancers including AGTR1 and ER-positive BCa. However, in light of the observation of ACE and AGTR1 polymorphisms, more population specific studies need to be carried out to fully understand the role of ACEi and ARBs with respect to anticancer therapy, with an ultimate goal of designing the framework for clinical trials and developing tailored treatment plan for cancer patients.
